# The Northern Ohio Dental Association

**Published:** 1891-07

**Authors:** 


### PROCEEDINGS.

### The Northern Ohio Dental Association.

The thirty-second annual meeting of the Northern Ohio Dental Association was held in the G. A. R. Hall, Oberlin, on Tuesday and Wednesday, May 12th and 13th, 1891.

This is the oldest organization except the Mississippi Valley Association. There are seventy-eight members in the association of which thirty-three were present. The name and residence of those in attendance is as follows:—C. R. Butler, Cleveland; Corydon Palmer, Warren; J. F. Siddall, Oberlin; F. S. Whitslar, Youngstown; W. H. Whitslar, Youngstown; Charles Buffett, Cleveland; J. E. Phelps, Chagrin Falls; Henry Barnes, Cleveland; Geo. H. Wilson, Painesville; H. F. Harvey, Cleveland; T. S. Seeley, Norwalk; G. R. Goulding, Cleveland; C. D. Peck, Sandusky; L. P. Bethel, Kent; W. H. Fowler, Painesville; F. H. Lyder, Akron; F. W. Knowlton, Akron; C. T. King, New London; F. F. Douds, Canton; J. F. Dougherty, Canton; J. H. Wible, Canton; Theo. J. Phillips, Canton; F. D. Davis, Minerva; D. S. Husted, Oberlin; H. G. Husted, Oberlin; Wm. F. Binsley, Napoleon; R. A. Dinsmore, Cleveland; W. A. Siddall, Oberlin; W. B. Connor, Akron; L. C. Kelsey, Elyria; A. W. Hazel, Elyria; Alfred Terry, Norwalk; Dr. Edson, Toledo.

Previous to the opening session of the meeting the members were escorted through the college buildings and shown every point of interest; in this way from the beginning to the end of the meeting all was business and constant attendance.

The meeting was called to order promptly at 10 A. M. by the President, Dr. F. S. Whitslar, of Youngstown. The reading of the minutes and all other business pertaining to opening a dental convention was disposed of by noon-time.

At 2 P. M. the session was opened by prayer by one of the leading ministers of the city.

The first essay on the programme was called for, and a paper on "Recurrence of Decay in Teeth," by J. G. Templeton, of Pittsburgh, was ordered read, Dr. Templeton being unable to attend. This paper was supplemented by a short paper on the same subject by Dr. F. S. Whitslar, of Youngstown. Both papers are to be found in the June REGISTER.

The discussion of the papers was opened by Dr. C. R. Butler, of Cleveland, as follows: "We are all interested in this subject because decay is the great enemy we have to fight. We can not take up the discussion of all the details of the subject because the same ground will have to be gone over that we perceive for primary decay, to a large extent. One of the many causes for recurrence of decay is the lapping over of the filling, this not having been dressed down to the margins of the cavity, a place for the lodgment of food is thus made and decomposition sets in with its consequences.

Many of the theories of decay have become obsolete. In reading the remarks of W. D. Miller, of Berlin, who is considered the best authority, the causes of decay have been settled, or his theories have been widely accepted, yet it must be admitted that there are chemical and chemico-vital forces at work as well as zymotic. As far as micro-organisms are concerned he is not willing to accept or admit that they bored into a tooth as do worms into wood, they don't work in that way. There was an attempt made in 1881 at the International Medical Congress, to show with some very beautiful slides, that micro-organisms go directly into enamel, but it is doubtful. Recurrence of decay is the great perplexity after filling teeth. There are some who say that teeth will not decay after being filled, but this is not true.

If we could manage the laws of the system to have a neutral condition of the fluids of the mouth we might, to a great extent, stop the decay of the teeth. It is retention of particles of food between the teeth, acid condition of the fluids of the mouth and vital forces depressed that cause the greatest trouble and recurrence is found most frequent. A proper system of cleansing helps much to prevent decay. There are conditions not explained satisfactorily in which fillings have been retained for years and then the tooth structure loses its integrity and breaks down. Recurrence in that locality no one can foresee.

Dr. Geo. H. Wilson.—One feature we can not be too particular about, and that is the shaping of cavities and contour of filling. Believe in capillary attraction. The actual point of contact of two teeth is not the actual place of decay, but rather below that, where the fluids are suspended. Do away with this point and better success may be attained.

In disease and old age we see a wasting away of tooth substance. Under such circumstances it is better to explain to the patients that conditions are unfavorable and they should not expect too much as we are laboring under conditions that are against us. Believes in perfect cleanliness and contoured fillings.

Dr. C. D. Peck.—Believes in gold as a filling material, but eight years ago introduced both gold and amalgam fillings in same mouth. Amalgam remained longer than the gold fillings. Wonders why?

Dr. W. H. Fowler.—Found in his earlier experience one great cause of recurrence of decay to be in putting his gold fillings in too well—he checked the enamel in introducing the filling.

Dr. T. S. Seely.—Said he would stick to the acid theory of decay yet. Believes all have failures and that causes may be overcome by cleansing cavity thoroughly, and good filling. Don't believe case is a failure if decay recurs in eight years.

Dr. R. A. Dinsmore.—Said much depends upon the structure of the teeth. Gold is not advisable in soft teeth. Cleanliness is a valuable point.

Dr. Chas. Buffett.—Said he failed to see the force of capillary attraction because the teeth are constantly bathed in fluids. Recurrence of decay may take place next to a filling that is well put in. Inflammation of the lungs may have an effect upon the teeth. Acid conditions, especially the nascent, is most powerful upon the teeth. Antiseptic mouth washes will do much good.

Dr. Geo. H. Wilson.—Capillary attraction, to his mind, has more to do with decay than we think of. When at rest, in thin places between the teeth, there is little chance for change, and little particles are held between the teeth unlike that upon flat surfaces.

Dr. Buffett asked, "If the mouth being constantly bathed with fluids, whether there will be capillary attraction?"

Dr. Wilson replied, "I think so."

Dr. H. F. Harvey.—Subject covers much latitude, takes in original cause, as it may be a recurrence after filling, or when decay seems to have stopped for a time. It may be recurrence or an occurrence. Contouring and also filing away may help to prevent recurrence. It is hard to draw the line between recurrence and original decay. Gestation and lactation may have much to do with decay.

Dr. Henry Barnes.—Reported having under his attention four tin fillings Dr. Atkinson put in fifty years ago. The fillings are gradually being worn away by attrition. Fillings are remarkable. He believes in capillary attraction.

Dr. Butler.—Said he did not wish the young men to think that the old members had attained that skill by which decay did not recur after their operations. Recurrence may be brought about by lack of judgment in prescribing the kind of filling to be used. Fees should have nothing to do with the case. Indifferent service given to the patient is the poorest kind of policy. Has been disgusted with men who have put gold into children's teeth when other filling material would do as well or better. Let the people understand we are masters of what we do.

Dr. T. S. Seeley.—Said it is as much of a difficulty to know what to use as to know how to put the filling in. We are tempted to do that which will bring the best remuneration.

Dr. W. H. Whitslar.—Said that one point brought out in Dr. Templeton's paper was well worth noticing, and that was, that failures in filling are most apt to occur along that margin of the cavity (lingual margin) which is next to the operator. In obscure cavities he found this point most difficult to fill always correctly. The subject was then passed.

The remaining part of the afternoon was devoted to the relation of incidents occurring during the lifetime of Dr. Wm. H.

Atkinson, with which Drs. Butler, Palmer, Buffett, Barnes and others were familiar, and concluded with an essay by Dr. J. F. Siddall, of Oberlin, whose subject was "As We Go Marching on." A part of this essay Dr. Siddall kindly consented to be published, and as it relates to Dr. Atkinson, it is of great interest and will be found in the next REGISTER.

The following was presented as the sense of the society on the deaths of

DR. WM. H. ATKINSON AND DR. JOHN STEPHAN.

By the irrevocable decree of The Ruler of the Universe the Northern Ohio Dental Association mourns the loss of one of it charter and honorary members Wm. H. Atkinson, M.D., D.D.S., having departed this life on April 2nd, 1891, in New York City.

Also, John Stephan, D.D.S., an active member who died June 25th, 1890, at Cleveland, Ohio.

In the decease of the former this society and the profession at large have lost one who was ever most keenly alive to the interests of the profession and a pioneer and leader in the science and art of dentistry.

In the death of the latter this society loses one of its most active and conscientious members.

C. R. BUTLER,
J. F. SIDDLE,
F. S. WHITSLAR,
CHAS. BUFFETT,
HENRY BARNES,
H. F. HARVEY,
Committee.

SESSION THURSDAY EVENING.

Meeting opened by President F. S. Whitslar.

An address of welcome was delivered by Prof. G. F. Wright, of Oberlin. It was a happy combination of wit and wisdom. He exhibited a mortar made of stone taken from Table mountain, out of a mine which had been run under a lava bed to the depth of 250 feet. The mortar is believed to be contemporaneous with the "Nampa Image" and the "Calveras skull" found in that region, and said to be not less than 15,000 years old. The address was highly entertaining and was fully appreciated by the association.

Addresses of welcome were also made by President Ballantine and Ex-President Fairchild, of the college, also editor Pierce, of the *Oberlin News.*

Responses were made by Drs. F. S. Whitslar and C. R. Butler. Dr. F. S. Whitslar then read his presidential address to the association which is published in the present number of the Register.

At mine o'clock the Obelin Glee Club entered the hall and entertained them till adjournment of the first day's session.

